# Genetic loss-of-function of activating transcription factor 3 but not C-type lectin member 5A prevents diabetic peripheral neuropathy

**DOI:** 10.1038/s41374-021-00630-5

**Published:** 2021-06-25

**Authors:** Hung-Wei Kan, Chin-Hong Chang, Ying-Shuang Chang, Yi-Ting Ko, Yu-Lin Hsieh

**Affiliations:** 1 School of Medicine for International Students, College of Medicine, I-Shou University, Kaohsiung, Taiwan; 2 Department of Surgery, Chi Mei Medical Center, Tainan, Taiwan; 3 Department of Anatomy, School of Medicine, College of Medicine, Kaohsiung Medical University, Kaohsiung, Taiwan; 4 School of Post-Baccalaureate Medicine, College of Medicine, Kaohsiung Medical University, Kaohsiung, Taiwan; 5 Department of Medical Research, Kaohsiung Medical University Hospital, Kaohsiung, Taiwan

**Keywords:** Chronic pain, Cellular neuroscience, Neuropathic pain

## Abstract

We investigated the mediating roles of activating transcription factor 3 (ATF3), an injury marker, or C-type lectin member 5A (CLEC5A), an inflammatory response molecule, in the induction of endoplasmic reticulum (ER) stress and neuroinflammation in diabetic peripheral neuropathy in ATF3 and CLEC5A genetic knockout (*aft3*^*−/−*^ and *clec5a*^*−/−*^, respectively) mice. ATF3 was expressed intranuclearly and was upregulated in mice with diabetic peripheral neuropathy (DN) and *clec5a*^*−/−*^ mice. The DN and *clec5a*^*−/−*^ groups also exhibited neuropathic behavior, but not in the *aft3*^*−/−*^ group. The upregulation profiles of cytoplasmic polyadenylation element-binding protein, a protein translation–regulating molecule, and the ER stress-related molecules of inositol-requiring enzyme 1α and phosphorylated eukaryotic initiation factor 2α in the DN and *clec5a*^*−/−*^ groups were correlated with neuropathic behavior. Ultrastructural evidence confirmed ER stress induction and neuroinflammation, including microglial enlargement and proinflammatory cytokine release, in the DN and *clec5a*^*−/−*^ mice. By contrast, the induction of ER stress and neuroinflammation did not occur in the *aft3*^*−/−*^ mice. Furthermore, the mRNA of reactive oxygen species–removing enzymes such as superoxide dismutase, heme oxygenase-1, and catalase were downregulated in the DN and *clec5a*^*−/−*^ groups but were not changed in the *aft3*^*−/−*^ group. Taken together, the results indicate that intraneuronal ATF3, but not CLEC5A, mediates the induction of ER stress and neuroinflammation associated with diabetic neuropathy.

## Abstract (web summary)

Neuroinflammation and endoplasmic reticulum stress is a critical process that leads to diabetic peripheral neuropathy (DPN). Activating transcription factor 3 (ATF3) is a susceptible molecule, and loss-of-function of ATF3, but not C-type lectin member 5A (CLEC5A), prevents the development of neuroinflammation and ER stress. Functional blockade of ATF3 may be a potential treatment for diabetic peripheral neuropathy by inhibiting neuroinflammation and cellular stress-induced ER stress.

## Introduction

Diabetic peripheral neuropathy is a complicated condition caused by hyperglycemia-induced endoplasmic reticulum (ER) stress [1] and neuroinflammation, and its treatment is a clinical challenge [2]. Diabetic peripheral neuropathy also shares the neuropathological characteristics of small-fiber neuropathy (SFN), such as reduction in the intraepidermal nerve fiber (IENF) density (skin denervation) and development of neuropathic behavior. Skin denervation after diabetes is correlated with neuropathic pain [3], indicating IENF densities as a predictor of diabetic peripheral neuropathy progression [4], and neuropathic behavior in diabetic patients [5–7]. However, why some diabetic patients with fewer IENFs experienced no pain during quantitative sensory testing remains unclear [8, 9]. The small nociceptors became sensitized after their terminal IENFs degeneration. Particularly, activating transcription factor 3 (ATF3), a potential neuronal marker under pathology, was activated on small nociceptors in concert with skin denervation [10], suggesting that ATF3 is a critical marker in addition to noxious transduction by IENF. ATF3 has also been suggested to be a marker of ER stress, and it negatively affects ER stress in obesity-related diabetes [11, 12] and in renal tissue failure [13] due to obesity-lipotoxicity-induced ATF3 activation and ER stress. ATF3 has paradoxical regulatory roles; ATF3 deficiency suppresses transplant rejection by ameliorating the inflammatory response [14]. By contrast, tissue-specific ATF3 deficiency exacerbates cardiomyopathy induced by obesity-related inflammation [15]. Moreover, ATF3 induction has been reported to promote inflammation [16] and to maintain energy homeostasis [17]. Despite the diverse roles of ATF3 in pathology and cellular homeostasis, the cascade signal between ER stress and ATF3 in diabetic peripheral neuropathy remains elusive. Furthermore, proinflammatory cytokines induce both ER stress and ATF3 upregulation [18], suggesting that diabetic peripheral neuropathy is a complicated neuropathology underlying neuroinflammation.

Neuroinflammation is microglia dependent, and microglial activation is associated with neuropathic pain [19]. C-type lectin member 5A (CLEC5A) is a critical molecule that mediates lethal inflammatory responses induced by dengue virus [20, 21], influenza viruses [22], Japanese encephalitis [23], and flavivirus [24]. CLEC5A is also involved in the pathogenesis of adult-onset Still’s disease [25] and dengue virus-activated osteoclast activation [26]. CLEC5A is expressed in immune cells, and CLEC5A activation triggers the burst release of proinflammatory cytokines, such as tumor necrosis factor-α (TNFα) [20, 27] and interleukin-6 (IL-6) [20]. Studies have demonstrated that in bacterial or viral infection, CLEC5A-expressing macrophages and microglia are required to induce lethal inflammation, which fails to occur under CLEC5A deficiency [20, 23, 24, 28]. The mechanisms underlying lethal inflammation include systemic microglial and macrophage activation, excessive inflammatory cytokine release [20, 23, 27], neutrophil extracellular trap formation [21, 24, 27], and platelet activation through exosome CLEC5A activation [21, 27]. However, the role of CLEC5A in diabetic peripheral neuropathy remains unclear. For example, whether CLEC5A is also a responding molecule that mediates diabetes-associated neuroinflammation is unknown. If it is not, the upstream molecule that modulates neuroinflammation and ER stress must be determined.

We used ATF3 (*aft3*^*−/−*^) and CLEC5A (*clec5a*^*−/−*^) genetic knockout mice to investigate the profiles of cytoplasmic polyadenylation element-binding protein (CPEB), a protein-translation-related molecule, and ER stress-related molecules of inositol-requiring enzyme 1α (IRE1α) and phosphorylated eukaryotic initiation factor 2α (p-eIF2α). The ultrastructural evidence obtained in this study verified the pathology of ER stress. The mRNA expression of TNFα (*Tnf-α*), IL-6 (*Il-6*), and reactive oxygen species (ROS)-removing enzymes, including superoxide dismutase (*Sod*), heme oxygenase-1 (*Ho-1*), and catalase (*Cat*), was also examined to elucidate the relationships among ATF3 activation, neuroinflammation, and ROS-removing in diabetic peripheral neuropathy. This study considered ATF3-mediated, but not CLEC5A-mediated, ER stress- and neuroinflammation-associated neuropathic manifestation in diabetic peripheral neuropathy.

## Materials and methods

### Diabetic peripheral neuropathy induction and animal groups

We used a modified diabetic peripheral neuropathy mouse model [29] with a single dose of streptozotocin (STZ, 200 mg/kg, Sigma, St. Louis, MO, USA) on three mouse strains: (1) 8-week-old C57/B6 mice, (2) age-matched *atf3*^*−/−*^ mice (gifted by Dr. Tsonwin Hai, The Ohio State University, Columbus, OH, USA), and (3) *clec5a*^*−/−*^ mice (gifted by Dr. Shie-Liang Hsieh, Genomic Research Center, Academia Sinica, Taiwan). The *atf3*^*−/−*^ and *clec5a*^*−/−*^ mice have a C57/B6 genetic background. The criteria of including in this study by mice exhibiting hyperglycemia (glucose level > 400 mg/dL) in 7 days after STZ treatment, and this study employed five groups: the (1) citrate (mice that received an equal volume of citrate solution, which served as the sham control), (2) DN (mice with glucose level > 400 mg/dL), (3) hypoDN (glucose level < 400 mg/dL, which served as the positive control), (4) *atf3*^*−/−*^, and (5) *clec5a*^*−/−*^ groups. The mice were housed in plastic cages under a 12-h light–dark cycle with access to food and water *ad libitum*. After neuropathic behavior evaluations at one month after treatment in each group, the mice were sacrificed with intracardiac perfusion, and the related tissues were harvested for subsequent pathological examinations. We made all possible efforts to minimize animal suffering and performed all procedures in a coded and blinded manner and in accordance with ethical guidelines related to laboratory animals. To confirm the genotypes of the *atf3*^*−/−*^ and *clec5a*^*−/−*^ mice used in this study, genotyping was performed through polymerase chain reaction (PCR) of the genomic DNA extracted from the tail. The same method employed and primer sequences in another study were used for *atf3* [10] and *clec5a* [20] genotyping.

### Neuropathic behavior evaluation

The activities and appearances of the mice were first evaluated before performing neuropathic behavior evaluation by assessing the thermal (hot-plate test) and mechanical (von Frey monofilament test) responses of the mice. We used the same methodology as that employed in a previous study [10]. For von Frey hair test [30], the up-and-down method was used [31] and the mechanical thresholds were calculated according to a published formula [32].

### Evaluation and quantitation of protein gene product 9.5(+) intraepidermal nerve fibers

IENFs were demonstrated by using a pan-axonal marker, protein gene product (PGP) 9.5, in immunohistochemical studies. Briefly, we employed anti-PGP9.5 (rabbit, 1:1000; UltraClone, Isle of Wight, UK) antiserum and the same methodology as that employed in another study [33].

### Investigation and quantification of different phenotypic dorsal root ganglia

We assessed the pathological profiles of the fourth- and fifth-lumbar dorsal root ganglia (DRG) by using a regular immunofluorescence approach. The primary antisera used were anti-ATF3 (rabbit, 1:200, Sigma), anti-NeuN (mouse, 1:200; Proteintech, Rosemont, IL, USA), anti-CPEB (goat, 1:200; Santa Cruz Biotechnology, Santa Cruz, CA, USA), anti-IRE1α (rabbit, 1:600; Abcam, Cambridge, MA, USA), anti-p-eIF2α (rabbit, 1:600; Thermo Fisher Scientific, Waltham, MA, USA), and anti–B cell lymphoma–extra large (Bcl-XL; rabbit, 1:250, Cell Signaling, Danvers, MA, USA) antisera. We employed the following combinations of primary antisera: (1) ATF3–NeuN, (2) CPEB–p-eIF2α, (3) CPEB–IRE1α, and (4) CPEB–Bcl-XL. For double labeling of ATF3–NeuN, the regular double-labeling immunostaining method was used, wherein antigen retrieval was conducted with citrate buffer at 95 °C for 30 min. The colocalized profiles were analyzed using ImageJ version 2.1.0 (National Institutes of Health, Bethesda, MD, USA) to estimate the Manders coefficient.

### Ultrastructural examinations of ER stress

We used DRG tissues to investigate the pathology of ER stress. Briefly, we dissected lumbar DRG tissues and postfixed the tissues in 2% osmium tetroxide for 2 h, dehydrated them through a graded ethanol series, and embedded them in Epon 812 resin (Polyscience, Philadelphia, PA, USA). Thin sections (50 nm) were stained with uranyl acetate and lead citrate, after which we observed them using an electron microscope (Hitachi, Tokyo, Japan) and photographed them.

### Investigation and quantitation of ionized calcium-binding adapter molecule 1 (Iba1)(+) microglia

We examined the pathologies of spinal microglia using an anti-Iba1 antiserum (rabbit, 1:1000; Wako, Osaka, Japan) with the immunostaining protocol; spinal cord tissues were subjected to cryosection, resulting in sections of 50-µm thickness. To ensure adequate sampling, we selected every sixth section of lumbar cord tissues, with six sections chosen in total. To quantify the Iba1(+) microglia, we measured the density, process number, and cell size of Iba1(+) microglia on laminae I and II of the dorsal horn [34]. The Iba1(+) microglial density was defined as the glia number divided by the dorsal horn area (glia/mm^2^).

### Reverse-transcription quantitative PCR (RT-qPCR) assessment of proinflammatory cytokines and ROS-removing enzymes

We extracted total RNA from each lumbar spinal cord by using NeucleoZOL (Macherey-Nagel, Dueren, Germany) and reverse transcribed the RNA using the ExcelRT Reverse Transcription Kit (SMBIO, Hsinchu, Taiwan) per the manufacturers’ protocols. This study used the IQ^2^ SYBR Green Fast qPCR System Master Mix LOW ROX kit (Bio-Genesis, Taipei, Taiwan) to perform the qPCR on QuantStudio 3 (Applied Biosystems, Waltham, MA, USA). Briefly, we conducted reactions in 96-well plates, and each sample was assayed in triplicate with the inclusion of a no-template control (no cDNA) for each primer set. The qPCR procedure comprised an initial polymerase activation at 95 °C for 2 min, followed by 40 cycles of denaturation at 95 °C for 5 s and annealing and extension at 58 °C for 20 s. We normalized the transcription levels of the target genes against those of glyceraldehyde-3-phosphate dehydrogenase (*Gapdh*) by using the 2^−ΔΔCt^ method. The gene-specific primer sequences used for the assessments were as follows: *Tnf-α*, sense 5′-AGCCGATTTGCTATCTCATACCAG-3′, and antisense 5′-CCTTCACAGAGCAATGACTCC-3′ [35]; *Il-6*, sense 5′-TTCCATCCAGTTGCCTTCTTG-3′, and antisense 5′-TTGGGAGTGGTATCCTCTGTGA-3′ [36]; *Sod*, sense 5′-CAATGGTGGGGGACATATTA-3′, and antisense 5′-TTGATAGCCTCCAGCAACTC-3′ [37]; *Ho-1*, sense 5′-CCTCACTGGCAGGAAATCATC-3′, and antisense 5′-CCTCGTGGAGACGCTTTACATA-3′ [28]; *Cat*, sense 5′-GAACGAGGAGGAGAGGAAAC-3′, and antisense 5′-TGAAATTCTTGACCGCTTTC-3′ [38]; and *Gapdh*, sense 5′-TTCCATCCTCCAGAAACCAG-3′, and antisense 5′-CCCTCGAACTAAGGGGAAAG-3′.

### Statistical analysis

To minimize individual variation, each group had 5–8 animals, and we express all data as the mean ± standard derivation of the mean; additionally, we performed one-way repeated-measures analysis of variance followed by Tukey’s *post hoc* test when *p* < 0.05, which indicated statistical significance.

## Results

### ATF3 was upregulated and colocalized with NeuN after STZ-induced diabetic peripheral neuropathy

**Fig. 1:**
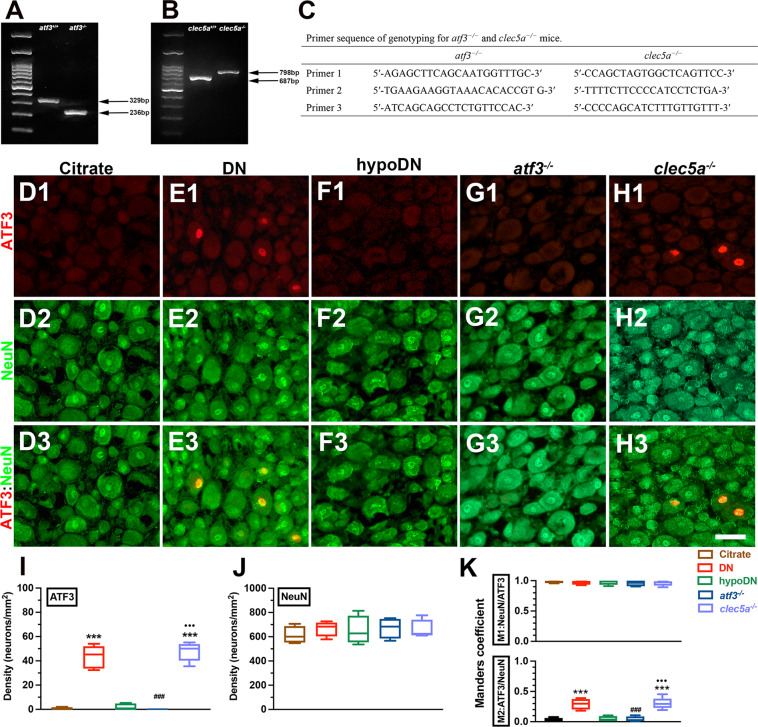
Genotypic confirmation and upregulation of activation transcription factor-3 (ATF3) after induction of diabetic peripheral neuropathy by streptozotocin (STZ). **A**–**C** The genotypes of ATF3 knockout (*atf3*^*−/−*^) and C-type lectin member 5A knockout (*clec5a*^*−/−*^) mice were confirmed using polymerase chain reaction (PCR) amplifications. **A** *atf3*^*−/−*^ mice showed 236 base-pair (bp) PCR products, and **B** *clec5a*^*−/−*^ mice had 798-bp PCR products. **C** Primer sequences used for genotyping in *atf3*^*−/−*^ and *clec5a*^*−/−*^ mice. **D**–**H** A mouse model of diabetic peripheral neuropathy was generated through intraperitoneal injection of STZ (200 mg/kg). Double-labeling immunofluorescent staining confirmed the intranuclear expression of ATF3 (**D**1–**H**1, red) colocalized with NeuN (**D**2–**H**2, green), a universal neuronal nuclear marker, in the dorsal root ganglion in the citrate (**D**1–**D**3), DN (blood glucose > 400 mg/dL; **E**1–**E**3), hypoDN (blood glucose < 400 mg/dL; **F**1–**F**3), *atf3*^*−/−*^ (**G**1–**G**3), and *clec5a*^*−/−*^ (**H**1–**H**3) groups at one month after STZ treatment. Photographs of ATF3(+) and NeuN(+) neurons (**D**3–**H**3) are merged for evaluating the Manders coefficient. Bar, 25 µm. Density changes in ATF3(+) (**I**) and (**J**) NeuN(+) neurons at each group. ATF3 was upregulated in DN and *clec5a*^*−/−*^ mice. *atf3*^*−/−*^ mice had no ATF3 expression, the NeuN(+) neuron densities were similar among the groups. **K** M1 (upper panel) and M2 (lower panel) Manders coefficients of ATF3(+) and NeuN(+) neurons according to colocalized patterns in Fig. 1D3–H3. Accordingly, the high NeuN signal and some nonspecific ATF3 signal resulted in a high M1 Manders coefficient, even after elimination of background signals in each group. The M2 Manders coefficient indicated ATF3 signal changes after DN. Group labels are indicated on each graph. ***p < 0.001: DN, hypoDN, *atf3*^*−/−*^, or *clec5a*^*−/−*^ group versus citrate group. ^###^*p* < 0.001: *atf3*^*−/−*^ or *clec5a*^*−/−*^ group versus DN group. •••*p* < 0.001: *atf3*^*−/−*^ versus *clec5a*^*−/−*^ group.

The genotypes of *atf3*^*−/−*^ (Fig. 1A) and *clec5a*^*−/−*^ (Fig. 1B) mice were confirmed through PCR amplification with related primer sequences (Fig. 1C), and only animals with 236 base-pair (bp) and 798 bp of PCR products were included in the *atf3*^*−/−*^ and *clec5a*^*−/−*^ groups, respectively. The expression of intranuclear ATF3 was confirmed through double-labeling immunostaining of ATF3 and NeuN, which are universal neuronal nuclear markers (Fig. 1D–H). Quantitatively, ATF3 was upregulated in DN (43.4 ± 9.4 vs. 0.73 ± 1.0 neurons/mm^2^, *p* < 0.001; Fig. 1E1–E3) and *clec5a*^*−/−*^ mice (47.6 ± 7.8 neurons/mm^2^, *p* < 0.001; Fig. 1H1–H3) compared with the citrate (Fig. 1D1–D3) and hypoDN groups (2.0 ± 2.7 neurons/mm^2^, *p* < 0.001; Fig. 1F1–F3). The *atf3*^*−/−*^ mice exhibited no ATF3 expression (Fig. 1G1–G3, 1I). Moreover, same densities of NeuN(+) neurons were observed in each group (*p* > 0.05; Fig. 1J). The colocalized Manders coefficients indicated that M2 ATF3(+)/NeuN(+) neuronal ratios were higher in the DN (0.29 ± 0.09, *p* < 0.001) and *clec5a*^*−/−*^(0.30 ± 0.09, *p* < 0.001) groups. However, M1 NeuN(+)/ATF3(+) neuronal ratios were determined by the high NeuN signal divided by the nonspecific ATF3 signal, although the background signal had been eliminated from each group (Fig. 1K).

### atf3^−/−^ mice exhibited no neuropathic behavior but presented skin denervation

**Fig. 2:**
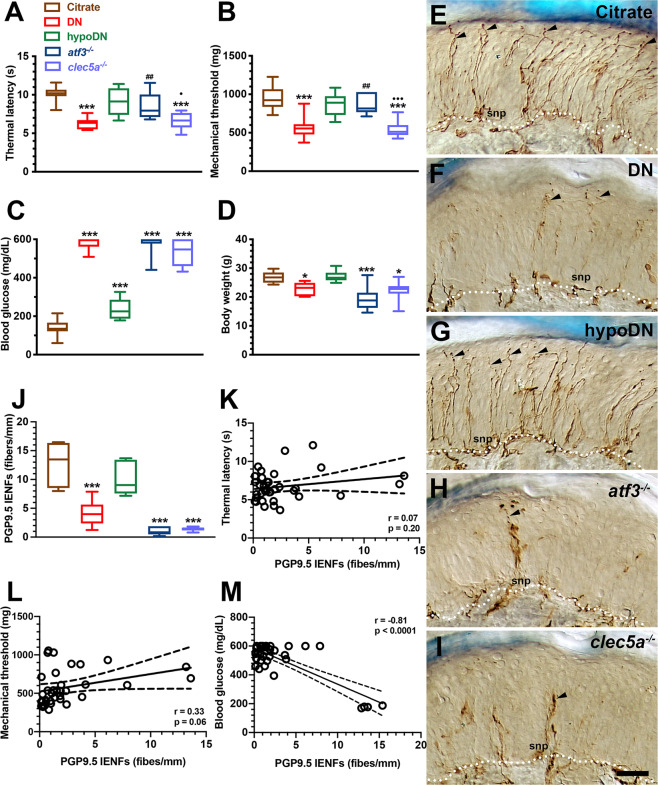
Development of neuropathic behavior and skin denervation in streptozotocin (STZ)-induced diabetic peripheral neuropathy. **A**–**D** After a diabetic peripheral neuropathy mouse model was generated with an intraperitoneal injection of STZ (200 mg/kg), neuropathic behaviors were evaluated using the hot-plate test (**A**) and von Frey hair test (**B**). Physiological examinations of blood glucose (**C**) and body weight (**D**) were performed on the citrate, DN (blood glucose > 400 mg/dL), hypoDN (blood glucose < 400 mg/dL) groups as well as two groups of transgenic mice—activating transcription factor 3 knockout (*atf3*^*−/−*^) and C-type lectin member 5A knockout (*clec5a*^*−/−*^) mice at one month after STZ treatment. Each group is labeled with a color box indicated in Fig. **A**. Changes in thermal latency (**A**) and mechanical threshold (**B**). DN and *clec5a*^*−/−*^ mice exhibited thermal hyperalgesia and mechanical allodynia; this neuropathic manifestation was absent in *atf3*^*−/−*^ mice. Changes in blood glucose (**C**) and body weight (**D**). *p < 0.05, ***p < 0.001: DN, hypoDN, *atf3*^*−/−*^, or *clec5a*^*−/−*^ group versus citrate group. ^##^*p* < 0.01: *atf3*^*−/−*^ or *clec5a*^*−/−*^ group versus DN group. •*p* < 0.05, •••*p* < 0.001: *atf3*^*−/−*^ versus *clec5a*^*−/−*^ group. **E**–**I** Intraepidermal nerve fibers (IENFs) visualized using the pan-axonal marker, protein gene product 9.5 (PGP9.5). PGP9.5(+) IENFs arise from the subepidermal nerve plexus (snp) and penetrate through the epidermal–dermal junction (white dashed line) with a typical varicose appearance (arrowhead). The profiles of PGP9.5(+) IENFs were markedly reduced in the DN (**F**), *atf3*^*−/−*^ (**H**), and *clec5a*^*−/−*^ (**I**) groups but not in the citrate (**E**) and hypoDN (**G**) groups. Bar, 50 µm. **J** Density changes of PGP9.5(+) IENFs according to Figs. **E**–**I**. Correlation of PGP9.5(+) IENF density with thermal latency (**K**), mechanical threshold (**L**), and blood glucose (**M**).

The DN mice exhibited both thermal hyperalgesia (6.2 ± 0.8 vs. 10.1 ± 1.0 s, *p* < 0.001) and mechanical allodynia (570.1 ± 140.7 vs. 953.0 ± 162.0 mg, *p* < 0.001) compared with the citrate and hypoDN groups (9.1 ± 1.7 s, *p* < 0.001 for thermal sensation and 874.4 ± 144.3 mg, *p* < 0.001 for mechanical sensation). The *clec5a*^*−/−*^ mice also exhibited thermal hyperalgesia (6.2 ± 1.1 s, *p* = 0.95) and mechanical allodynia (539.4 ± 107.8 mg, *p* = 0.99). Notably, these neuropathic behaviors were absent in the *atf3*^*−/−*^ mice (8.5 ± 1.7 s, *p* = 0.07 for thermal sensation and 834.8 ± 144.5 mg, *p* = 0.70 for mechanical sensation) compared with the citrate group (Fig. 2A, B). However, the DN, *atf3*^*−/−*^, and *clec5a*^*−/−*^ mice exhibited similar hyperglycemia (Fig. 2C) and body weight loss (Fig. 2D) compared with the mice in the citrate and hypoDN groups.

Notably, the degree of skin innervation was uncorrelated with neuropathic behavior. For example, PGP9.5(+) IENFs emerged from the subepidermal nerve plexuses (snp in Fig. 2E–I), with a typical varicose appearance (arrowhead in Fig. 2E–I), and the density of PGP9.5(+) IENFs was lower in the DN (4.1 ± 2.1 fibers/mm; *p* < 0.001), *atf3*^*−/−*^ (1.0 ± 0.7 fibers/mm; *p* < 0.001), and *clec5a*^*−/−*^ (1.4 ± 0.3 fibers/mm; *p* < 0.001) groups than in the citrate (12.6 ± 3.9 fibers/mm) and hypoDN groups (10.2 ± 3.0 fibers/mm; Fig. 2E–J). The linear correlation analyses further confirmed that the thermal latency (*r* = 0.07, *p* = 0.20; Fig. 2K) and mechanical threshold (*r* = 0.33, *p* = 0.06; Fig. 2L) were independent to PGP9.5(+) IENF density. By contrast, the PGP9.5(+) IENF density was linearly correlated with blood glucose (*r* = −0.81, *p* < 0.0001; Fig. 2M).

### Coexpression of CPEB(+) and ER stress-related molecules was correlated with a neuropathic manifestation after diabetic peripheral neuropathy induction

**Fig. 3:**
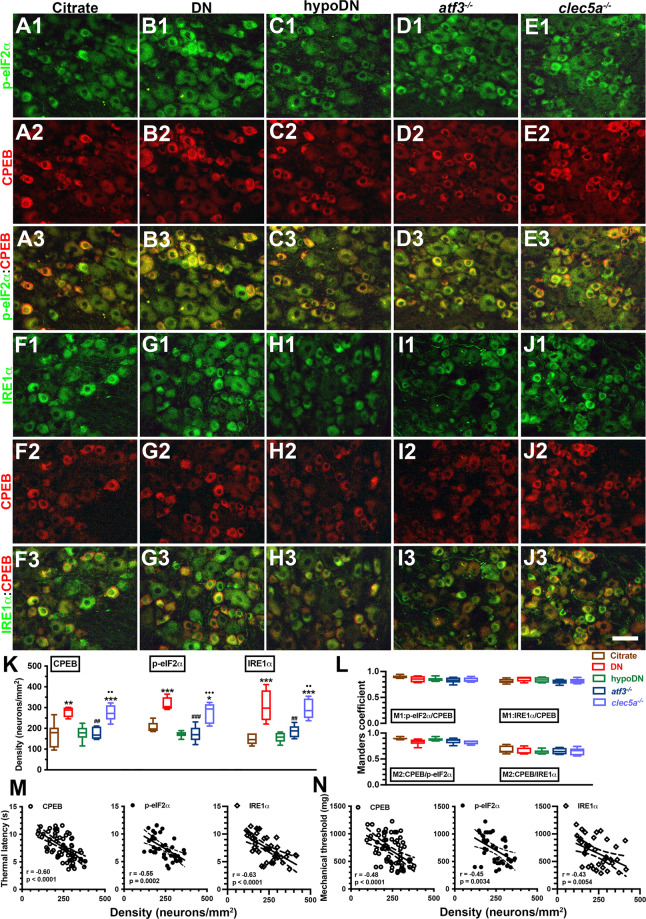
Colocalization of endoplasmic reticulum (ER) stress-related molecules and cytoplasmic polyadenylation element-binding protein (CPEB)(+) neurons in streptozotocin (STZ)-induced diabetic peripheral neuropathy. Double-labeling immunofluorescent staining was performed in two combinations—(1) phosphorylated eukaryotic initiation factor 2α (p-eIF2α) (**A**1–**E**1, green) with CPEB (**A**2–**E**2, red) and (2) inositol-requiring enzyme 1α (IRE1α) (**F**1–**J**1, green) with CPEB (**F**2–**J**2, red)—in the dorsal root ganglion of the citrate (**A**1–**A**3 and **F**1–**F**3), DN (blood glucose > 400 mg/dL; **B**1–**B**3 and **G**1–**G**3), hypoDN (blood glucose < 400 mg/dL; **C**1–**C**3 and **H**1–**H**3), activating transcription factor 3 knockout (*atf3*^*−/−*^; **D**1–**D**3 and **I**1–**I**3), and C-type lectin member 5A knockout (*clec5a*^*−/−*^; **E**1–**E**3 and **J**1–**J**3) groups at one month after STZ treatment. Photographs of p-eIF2α(+) and CPEB(+) neurons (**A**3–**E**3) as well as IRE1α(+) and CPEB(+) neurons (**F**3–**J**3) are merged to enable efficient analysis of the Manders coefficient colocalization. **A**–**J** ER stress-related molecules, p-eIF2α(+) and IRE1α(+), and CPEB(+) neurons were increased in the DN group but were at normal levels in the *atf3*^*−/−*^ group; no normalization effect was discovered in the *clec5a*^*−/−*^ group for these molecules. Bar, 25 µm. Density changes of CPEB (left panel), p-eIF2α (middle panel), and IRE1α (right panel) neurons (**K**) and (**L**) Manders coefficients M1 (upper panel) and M2 (lower panel) of p-eIF2α(+) and CPEB(+), and IRE1α(+) and CPEB(+) neurons according to colocalized patterns in Fig. 3A3–E3 and F3–J3. Group labels are indicated on each graph. *p < 0.05, **p < 0.01, ***p < 0.001: DN, hypoDN, *atf3*^*−/−*^, or *clec5a*^*−/−*^ group versus citrate group. ^##^*p* < 0.01, ^###^*p* < 0.001: *atf3*^*−/−*^ or *clec5a*^*−/−*^ group versus DN group. ••*p* < 0.01, •••*p* < 0.001: *atf3*^*−/−*^ versus *clec5a*^*−/−*^ group. Densities of CPEB(+) (open circles), p-eIF2α(+) (closed circles), and IRE1α(+) (open diamonds) neurons are inversely to the changes of thermal latency (**M**) and mechanical threshold (**N**).

CPEB regulates protein translation, which is essential for pain development [38]. Therefore, we investigated the coexpression pattern of CPEB with the ER stress-related molecules of p-eIF2α and IRE1α (Fig. 3). CPEB and p-eIF2α were preferentially expressed by small-diameter neurons, and these CPEB(+) neurons (280.8 ± 25.4 vs. 168.9 ± 59.2 neurons/mm^2^, *p* = 0.0024) and p-eIF2α(+) neurons (316.3 ± 32.9 vs. 205.7 ± 24.4 neurons/mm^2^, *p* = 0.0003) were upregulated in the DN group compared with the citrate and hypoDN groups (176.1 ± 36.5 neurons/mm^2^, *p* = 0.004 for CPEB; 172.2 ± 37.8 neurons/mm^2^, *p* < 0.001 for p-eIF2α; Fig. 3K). In the *atf3*^*−/−*^ mice, there were no upregulated patterns of CPEB (175.1 ± 29.0 neurons/mm^2^, *p* = 0.001) and p-eIF2α (172.2 ± 14.2 neurons/mm^2^, *p* < 0.001; Fig. 3D1–D3). However, the *clec5a*^*−/−*^ mice showed similar CPEB (296.7 ± 44.2 neurons/mm^2^, *p* = 1.00) and p-eIF2α (273.6 ± 49.8 neurons/mm^2^, *p* = 0.34; Fig. 3E1–E3) expression patterns as those in the DN group. Furthermore, similar patterns were found for IRE1α, another ER stress-related molecule (Fig. 3F1–J3, K). The colocalized Manders coefficients revealed that M1 p-eIF2α/CPEB (0.83–0.90) and M1 IRE1α/CPEB (0.80–0.86) expression was similar in each group. The M2 Manders coefficients revealed an overlap in CPEB/p-eIF2α (0.82–0.90) and CPEB/IRE1α (0.63–0.68; Fig. 3L), indicating that CPEB was highly colocalized with ER stress-related molecules. Furthermore, the CPEB, p-eIF2α, and IRE1α densities were inversely correlated with the thermal latency (Fig. 3M) and mechanical threshold (Fig. 3N).

**Fig. 4:**
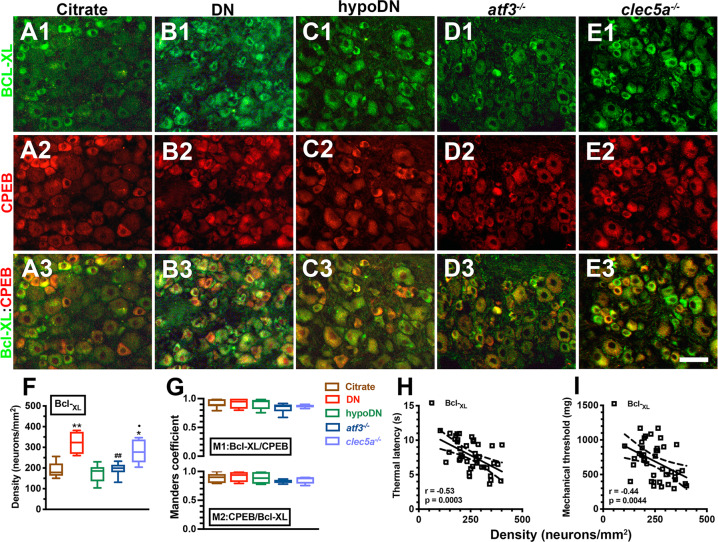
Coexpression patterns of B cell lymphoma–extra large (Bcl-XL)(+)/CPEB(+) neurons after streptozotocin (STZ)-induction of diabetic peripheral neuropathy. Double-labeling immunofluorescent staining was performed with anti-Bcl-XL (**A**1–**E**1, green) and anti-CPEB (**A**2–**E**2, red) in the dorsal root ganglion in the citrate (**A**1–**A**3), DN (blood glucose > 400 mg/dL; **B**1–**B**3), hypoDN (blood glucose < 400 mg/dL; **C**1–**C**3), activating transcription factor 3 knockout (*atf3*^*−/−*^; **D**1–**D**3), and C-type lectin member 5A knockout (*clec5a*^*−/−*^; **E**1–**E**3) groups at one month after STZ treatment. Photographs of Bcl-XL(+) and CPEB(+) neurons (**A**3–**E**3) are merged to enable efficient analysis of the Manders coefficient colocalization. Bar, 25 µm. Density changes of Bcl-XL(+) (**F**) and (**G**) Manders coefficients M1 (upper panel) and M2 (lower panel) of Bcl-XL(+) and CPEB(+) neurons according to colocalized patterns in Fig. 4A3–E3. The upregulation of Bcl-XL(+) neurons was observed in DN and *clec5a*^*−/−*^ mice, but not in *atf3*^*−/−*^ mice. Group labels are indicated on each graph. *p < 0.05, **p < 0.01: DN, hypoDN, *atf3*^*−/−*^, or *clec5a*^*−/−*^ group versus citrate group. ^##^*p* < 0.01: *atf3*^*−/−*^ or *clec5a*^*−/−*^ group versus DN group. •*p* < 0.05: *atf3*^*−/−*^ versus *clec5a*^*−/−*^ group. Density of Bcl-XL(+) neurons is inversely to the changes of thermal latency (**H**) and mechanical threshold (**I**).

Because Bcl-XL is responsible for cellular stress [39], we then investigated the change in the co-expressed profile of CPEB(+) and Bcl-XL(+) neurons (Fig. 4). We mostly detected Bcl-XL expression in the small-diameter neurons exhibiting upregulation in the DN (Fig. 4B1–B3; *p* = 0.003) and *clec5a*^*−/−*^ (Fig. 4E1–E3; *p* = 0.03) groups but not in the *atf3*^*−/−*^ group (Fig. 4D1–D3; *p* = 0.99) compared with the citrate and hypoDN groups (Fig. 4F). The colocalized M1 Bcl-XL(+)/CPEB(+) (0.84–0.92) and M2 CPEB(+)/Bcl-XL(+) (0.83–0.92) showed a high overlap between Bcl-XL and CPEB among all groups (Fig. 4G). In addition, the Bcl-XL(+) neuron density was also inversely correlated with the thermal latency (*r* = −0.53, *p* = 0.0003; Fig. 4H) and mechanical threshold (*r* = −0.44, *p* = 0.0044; Fig. 4I). Collectively, the CPEB expression profile paralleled the alteration of ER stress-related molecules, such as p-eIF2α, IRE1α, and Bcl-XL, which are all related to the development of neuropathic behaviors.

### ER stress was prevented in atf3^−/−^ mice after STZ-induced diabetic peripheral neuropathy

**Fig. 5:**
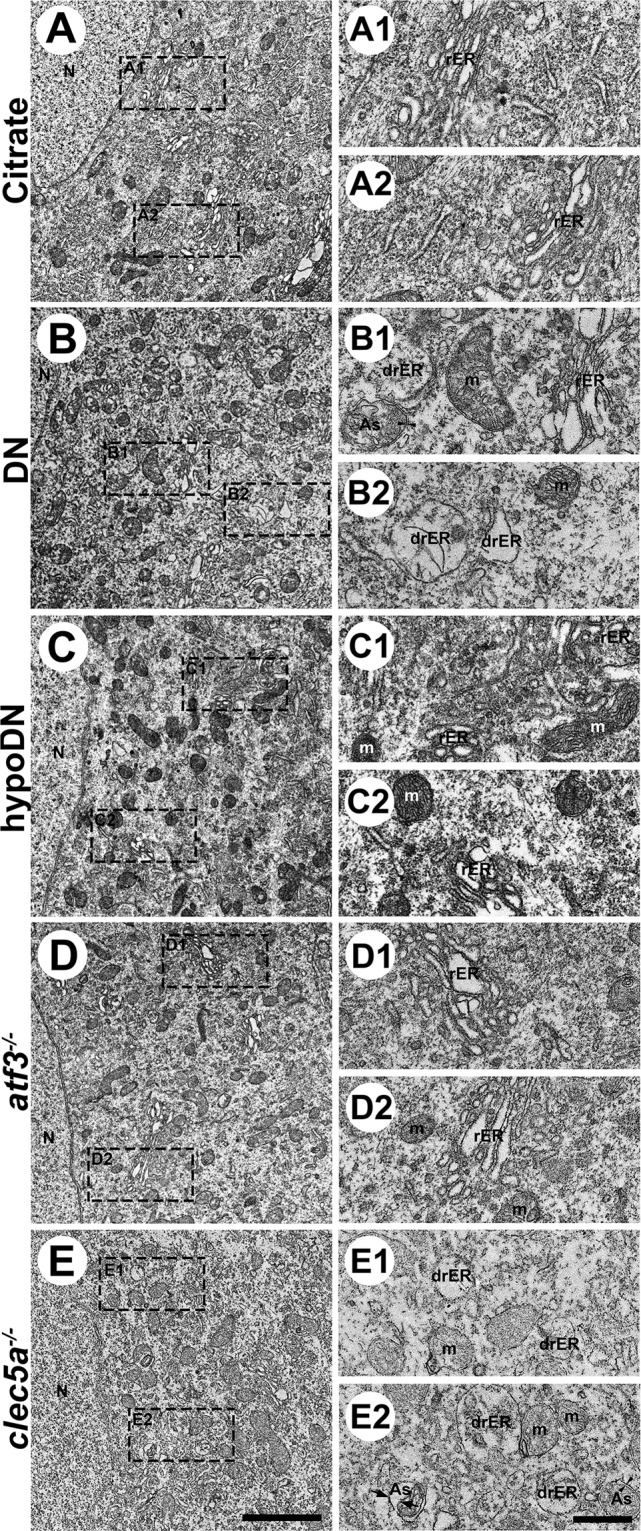
Ultrastructural examination of the ER in streptozotocin (STZ)-induced diabetic peripheral neuropathy. The lumbar dorsal root ganglia of the citrate (**A**), DN (blood glucose > 400 mg/dL; **B**), hypoDN (blood glucose < 400 mg/dL; **C**), activating transcription factor 3 knockout (*atf3*^*−/−*^; **D**), and C-type lectin member 5A knockout (*clec5a*^*−/−*^; **E**) groups at one month after STZ treatment, with samples prepared for electron microscopy examinations. Ultrastructural examinations of the ER (5000×) next to the cell nucleus (N; **A**–**E**), which is ER-rich, were performed. Bar, 1 µm. (**A**1–**E**2) Higher magnification (15000×) in the insets. The ER in the citrate (**A**1, **A**2) and hypoDN (**C**1, **C**2) groups appeared as a stack of flattened membrane-bound cisternae with a lucent lumen. The *atf3*^*−/−*^ mice had a similar ER ultrastructural profile with some dilated lucent ER lumen (**D**1, **D**2). By contrast, the DN (**B**1, **B**2) and *clec5a*^*−/−*^ (**E**1, **E**2) groups had dilated ER lumen (drER) that contained amorphous or granular substances. Some autophagosomes with a double limiting membrane (arrowheads in B1 and E2) were also observed in the DN and *clec5a*^*−/−*^ groups. Other autophagosomes had a double limiting membrane with an away-from-each-other fashion (arrows in **E**2). Bar, 250 nm. m mitochondria, rER rough endoplasmic reticulum, drER dilated rough endoplasmic reticulum, As autophagosome.

Proper protein folding and assembly in rough ER (rER) lumen is crucial for normal cellular physiology; thus, we performed ER ultrastructural examinations of DRGs to confirm the altered profile of ER stress-related molecules indicated by the immunostaining study (Fig. 5). Regarding its ultrastructural morphology, the rER comprised stacks of flattened membrane-bound cisternae with a lucent lumen in the citrate (Fig. 5A1, A2) and hypoDN (Fig. 5C1, C2) groups. By contrast, the rER morphology in the DN group exhibited the typical characteristics of ER stress, such as dilated rER (drER in Fig. 5B1, B2) lumen with amorphous masses implying the accumulation of misfolded or unfolded proteins; additionally, we observed some autophagosomes with double limiting membrane [40] (As in Fig. 5B1) within DRG soma (Fig. 5B1, B2). The *atf3*^*−/−*^ mice exhibited less ER stress; that is, the rER comprised normal stacks with flattened membrane-bound and clear lumen, with only minimal rER having slightly dilated lumen (Fig. 5D1, D2). By contrast, the *clec5a*^*−/−*^ mice had dilated rER lumen with amorphous materials and double limiting membrane autophagosome (drER and As in Fig. 5E1, E2).

**Fig. 6:**
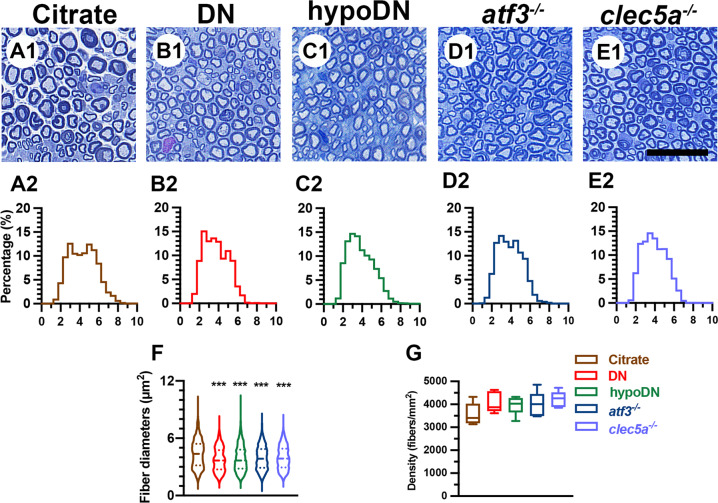
Morphometry of the sural nerve after streptozotocin (STZ) was used to induce diabetic peripheral neuropathy. Photographs showing semithin sections of epon-embedded sural nerves from the citrate (**A**1), DN (blood glucose > 400 mg/dL; **B**1), hypoDN (blood glucose < 400 mg/dL; **C**1), activating transcription factor 3 knockout (*atf3*^*−/−*^; **D**1), and C-type lectin member 5A knockout (*clec5a*^*−/−*^; **E**1) groups at one month after STZ treatment. Morphometric analyses of sural nerve reveal the (1) distribution of fiber diameters, represented as a spectrum histogram (**A**2–**E**2) and violin box (**F**) and (2) the density changes of myelinated sural nerve fibers (**G**). The distribution of fiber diameters for the sural nerve was bimodal for the citrate group but unimodal for the DN, hypoDN, *atf3*^*−/−*^, and *clec5a*^*−/−*^ groups.

To determine whether the pathology of myelinated sural sensory nerve contributed to neuropathic behavior, we investigated the morphometry of the sural nerves (Fig. 6). The citrate group had large- and small-diameter populations of myelinated sensory nerves, resulting in a bimodal histogram (Fig. 6A1, A2). However, the profile was a unimodal histogram for the DN, hypoDN, *atf3*^*−/−*^, and *clec5a*^*−/−*^ groups (Fig. 6B1–E2). The mean fiber diameter of the citrate group was larger (4.4 ± 1.4 µm, 25th–75th percentile: 3.2–5.4 µm; *p* < 0.001) than that of the DN (3.8 ± 1.2 µm, 25th–75th percentile: 2.7–4.8 µm; *p* < 0.001), hypoDN (3.9 ± 1.4 µm, 25th–75th percentile: 2.8–4.8 µm; *p* < 0.001), *atf3*^*−/−*^ (3.9 ± 1.2 µm, 25th–75th percentile: 2.9–4.9 µm; *p* < 0.001), and *clec5a*^*−/−*^ (4.0 ± 1.2 µm, 25th–75th percentile: 2.9–4.9 µm; *p* < 0.001) groups (Fig. 6F). Moreover, the density of myelinated fibers in the sural nerves was indistinguishable between all groups (Fig. 6G; *p* > 0.05). These findings suggested ER stress induction related to diabetic peripheral neuropathy.

### atf3^−/−^ mice prevent neuroinflammation after STZ-induced diabetic peripheral neuropathy

**Fig. 7:**
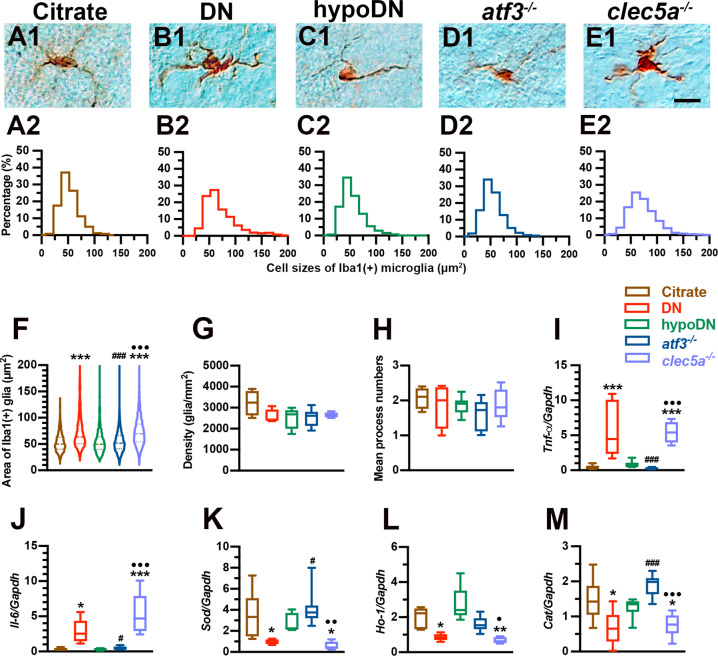
Absence of microglia activation and neuroinflammation in the *atf3*^*−/−*^ group after streptozotocin (STZ) induction of diabetic peripheral neuropathy. Microglia on the lumbar cord are indicated by anti-ionized calcium-binding adapter molecule 1 (Iba1) antisera through immunostaining in the citrate (**A**1), DN (blood glucose > 400 mg/dL; **B**1), hypoDN (blood glucose < 400 mg/dL; **C**1), activating transcription factor 3 knockout (*atf3*^*−/−*^; **D**1), and C-type lectin member 5A knockout (*clec5a*^*−/−*^; **E**1) groups at one month after treatment. Bar, 25 µm. Quantification is performed using the (1) size, (2) density, and (3) mean process number of Iba1(+) microglia. Histograms showing the frequency ratios of Iba1(+) microglial size in the citrate (**A**2), DN (**B**2), hypoDN (**C**2), *atf3*^*−/−*^ (**D**2), and *clec5a*^*−/−*^ (**E**2) groups, quantified using the respective figure in Figs. **A**1–**E**1. **F** Distribution of Iba1(+) microglia size as well as the median (dashed line) and 25% and 75% percentiles (dotted lines). The DN and *clec5a*^*−/−*^ groups had enlarged microglia, indicating that the Iba1(+) microglia were activated. Density (**G**) and mean process changes (**H**) of Iba1(+) microglia on laminae I and II of the dorsal horn in the citrate, DN, hypoDN, *atf3*^*−/−*^, and *clec5a*^*−/−*^ groups. No intergroup differences were discovered in density or mean process of Iba1(+) microglia. Expression changes of tumor necrosis factor-α (*Tnf-α*) (**I**), interleukin-6 (*Il-6*) (**J**), superoxide dismutase (*Sod*) (**K**), heme oxygenase-1 (*Ho-1*) (**L**), and catalase (*Cat*) (**M**) mRNA assayed using quantitative PCR normalized to glyceraldehyde-3-phosphate dehydrogenase (*Gapdh*). *Tnf-α* and *Il-6* were increased in the DN and *clec5a*^*−/−*^ groups, whereas *Sod*, *Ho-1* and *Cat* levels in the *atf3*^*−/−*^ group were higher than those in the *clec5a*^*−/−*^ group. Group labels are indicated on each graph. *p < 0.05, **p < 0.01, ***p < 0.001: DN, hypoDN, *atf3*^*−/−*^, or *clec5a*^*−/−*^ group versus citrate group. ^#^*p* < 0.05, ^###^*p* < 0.001: *atf3*^*−/−*^ or *clec5a*^*−/−*^ group versus DN group. •*p* < 0.05, ••*p* < 0.01, •••*p* < 0.001: *atf3*^*−/−*^ versus *clec5a*^*−/−*^ group.

To investigate the role of ATF3 and CLEC5A, we examined the induction of microglia pathology and burst release of proinflammatory cytokines. Iba1(+) microglia on lumbar cords had a small oval cell body profile in the citrate and hypoDN groups (Fig. 7A1 and C1, respectively), whereas the cell body of Iba1(+) microglia in the DN group was larger (Fig. 7B1). Quantitatively, the histogram of cell size for the DN group was right-shifted compared with that for the citrate and hypoDN groups (Fig. 7A2–C2). The Iba1(+) microglia profile in *atf3*^*−/−*^ mice revealed small cells (Fig. 7D1), but an increase in the Iba1(+) microglia profile was discovered in *clec5a*^*−/−*^ mice (Fig. 7E1 and D2–E2). The DN group had a larger mean cell size (73.5 ± 35.7 µm^2^, 25th–75th percentile: 50.8–85.4 µm^2^) compared with the citrate (53.1 ± 17.8 µm^2^, 25th–75th percentile: 40.2–62.6 µm^2^, *p* < 0.001) and hypoDN (54.4 ± 24.4 µm^2^, 25th–75th percentile: 39.7–63.5 µm^2^, *p* < 0.001) groups. The *atf3*^*−/−*^ mice had smaller mean cell size (55.1 ± 21.0 µm^2^, 25th–75th percentile: 41.0–65.5 µm^2^) compared with the *clec5a*^*−/−*^ mice (73.8 ± 28.9 µm^2^, 25th–75th percentile: 54.1–88.1 µm^2^, *p* < 0.001; Fig. 7F). We discovered no intergroup differences in density (*p* > 0.05; Fig. 7G) or in the mean process numbers of Iba1(+) microglia (*p* > 0.05; Fig. 7H).

In the RT-qPCR assessments (Fig. 7I–M), we discovered that *Tnf-α* (Fig. 7I) and *Il*−6 (Fig. 7J) mRNA expressions were upregulated 14.0-fold (*p* < 0.001) and 9.8-fold (*p* < 0.001), respectively, in the DN group; by contrast, *Sod* (Fig. 7K), *Ho-1* (Fig. 7L), and *Cat* (Fig. 7M) mRNA expressions were downregulated 3.6-fold (*p* = 0.02), 2.3-fold (*p* = 0.01), and 2.2-fold (*p* = 0.003), respectively. The *clec5a*^*−/−*^ mice had similar mRNA expression patterns with the DN group; that is, *Tnf-α* and *Il-6* mRNA expressions were upregulated 12.9-fold (*p* < 0.001) and 17.5-fold (*p* < 0.001), respectively, and *Sod*, *Ho-1*, and *Cat* mRNA expressions were downregulated 6.1-fold (*p* = 0.01), 3.0-fold (*p* = 0.003), and 1.97-fold (*p* = 0.02), respectively. However, these mRNA expression patterns were not observed in the *atf3*^*−/−*^ mice. Notably, this gene expression of proinflammatory cytokines and ROS-removing enzymes in lumbar cord tissues was related to neuropathic behavior.

## Discussion

### Application of IENF assessment in clinical diagnosis of diabetic peripheral neuropathy and unresolved issues

The assessment of the IENF density through the combination of skin biopsy and PGP 9.5 immunohistochemistry is a reliable clinical diagnostic tool and can be used to predict neuropathy progression [4]. However, the present study demonstrated that the reduced IENF density after diabetic peripheral neuropathy was hyperglycemia-dependent but was independent of neuropathic manifestation. The evaluation of neuropathic manifestation in patients with diabetic peripheral neuropathy requires different approaches [8], and because it is a systemic metabolic disorder, the underlying mechanism may involve different neuronal-related cells [41]. Our previous study demonstrated that ATF3 expression by the neuronal ε isoform of protein kinase determined neuropathic behavior in a mouse model of diabetic peripheral neuropathy [10]. The current finding further demonstrated the participation of ATF3 in intranuclear signal modulation by the genetic deletion of ATF3 resulting in the reduction of neuroinflammation and ER stress without protective effects on skin denervation, suggesting that the neuronal soma may be a critical response center for nociceptive modulation than for nociceptive transduction by IENFs.

### Neuronal injury-dependent ATF3 upregulation mediated neuroinflammation and ER stress

ATF3 is a susceptible intranuclear molecule associated with neuropathic manifestation in diabetic peripheral neuropathy [10], and injury-dependent ATF3 upregulation is associated with neuroinflammation [42], implying that neuroinflammation is a critical neuropathological process underlying pain development [43]. The present study demonstrated intranuclear ATF3 upregulation only in DN and *clec5a*^*−/−*^ mice, which is associated with neuropathic manifestation, ER stress, and neuroinflammation induction. Although CLEC5A is essential for inflammatory responses [20, 23, 24, 28], this study revealed that CLEC5A molecules are not required for diabetic peripheral neuropathy development because microglial activation and proinflammatory cytokine release still occurred in *clec5a*^*−/−*^ mice. Proinflammatory cytokines induce ATF3 upregulation and ER stress [18]. The present study revealed a further link between ATF3 upregulation and ER stress induction; in particular, this study demonstrated that ATF3 mediated both neuroinflammatory responses and ER stress induction. For example, these neuroinflammation-related pathologies and ER stress were not observed in *atf3*^*−/−*^ mice, suggesting that ATF3 plays a more crucial role than CLEC5A in neuroinflammation mediation (i.e., there is an intraneuronal response [42] rather than an extracellular ligand–induced systemic inflammatory response).

CPEB is synthesized following ER stress due to fatty-liver-related inflammation [44] and is responsible for pain development [38]. CPEB regulated the inflammatory response through intranuclear signaling [45]. This current study revealed the co-upregulation of CPEB and the ER stress-related molecules of p-eIF2α and IRE1α in DN and *clec5a*^*−/−*^ mice, but not in *atf3*^*−/−*^ mice, suggesting that the pathological profiles of these molecules are regulated by neuronal injury-dependent neuroinflammation. Under injury stress, Bcl-XL, an antiapoptotic Bcl 2 family protein, has a neuroprotective effect [39]; the present study also demonstrated the co-upregulation pattern of CPEB and Bcl-XL under neuronal injury. These physiological linkages may be attributed to CPEB aggregation–induced cellular stress, resulting in protein disassembly–induced neuropathy [46]. In addition, CPEB knockdown with antisense oligodeoxynucleotide relieved neuropathic pain [47]. Recently, our research group demonstrated a reduction in Bcl-XL upregulation in *atf3*^*−/−*^ mice after diabetic peripheral neuropathy [10], and the current study further revealed that *atf3*^*−/−*^ mice did not exhibit the co-upregulation of CPEB and Bcl-XL. Importantly, this is first study to suggest that ATF3 regulates CPEB pathophysiological activities under injury stress; that is, these pieces of evidence suggest that ATF3 is an upstream modulator responsible for neuronal injury-dependent neuroinflammation and ER stress, which lead to a neuropathic manifestation in diabetic peripheral neuropathy.

### Importance and implications of ATF3-modulated cellular oxidative stress

**Fig. 8:**
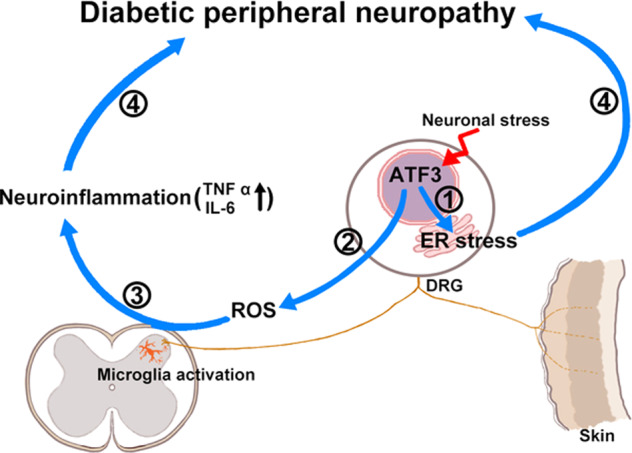
Mechanisms of intranuclear ATF3-mediated diabetic peripheral neuropathy. ATF3, a susceptible molecule, is activated under neuronal stress. ATF3 mediated (1) the induction of ER stress pathology and (2) ROS production, which induced (3) neuroinflammation including microglial activation and increase in TNFα and IL-6 release. Both ER stress induction and neuroinflammation resulted in the evoking of diabetic peripheral neuropathy. ATF3 activating transcription factor 3, DRG dorsal root ganglion, ROS reactive oxygen species, TNFα tumor necrosis factor α, IL-6 interleukin-6.

ER stress and neuroinflammation are the major pathologies of obesity-associated diabetes [11, 12] under lipotoxic-dependent diabetic neuropathy. In the current mouse model of diabetic peripheral neuropathy induced by STZ, β-islet cells, which mimic insulin-dependent and obesity-independent diabetes, were destroyed. Accordingly, ER stress and neuroinflammation were also induced through a lipotoxic-independent pathway, which may be injury-dependent [48]. Although ER stress induction (in DRGs) and neuroinflammation (in the lumbar spinal cord) are locally distinct, ROS could be used as an intermediate between injured neurons and activated microglia due to ROS attenuation, which reduced the neuropathic manifestation [49]. The relationships of ATF3 and ROS have been investigated; for example, the loss of ROS scavenger correlated to ATF3 upregulation, and ATF3 deficiency could against bacterial and fungal infection even under ROS stress [50]. In addition, ATF3 silencing by small interfering RNA enhanced the antioxidant effect and attenuated ER stress [11] as well as prevented ER stress-associated cell death [51], suggesting that ATF3 mediates oxidative stress [52]. This ATF3-dependent oxidative stress involved ROS production and inflammation during injury [53, 54]. The present study confirmed that the genetic expression of ROS-removing enzyme, *Sod*, *Ho-1*, and *Cat* mRNA was downregulated by DN and *clec5a*^*−/−*^, but this was no changed in the *atf3*^*−/−*^ mice. Accordingly, this study employed two genetic knockout mice, *atf3*^*−/−*^ and *clec5a*^*−/−*^ mice, to demonstrate that ATF3 is a crucial intraneuronal upstream molecule such as the genetic loss-of-function of ATF3 that not only prevents intracellular ER stress signals but also eliminates ROS by maintaining a steady level of the ROS-removing enzyme. This pathology of ER stress and excessive ROS were believed to induce the diabetic peripheral neuropathy (Fig. 8). Collectively, the evidence furnished by this study suggests ATF3’s potential as a therapeutic target for neuropathic pain control in lipotoxic-independent diabetic peripheral neuropathy.

## Data Availability

The data that support the findings of this study are available on request from the corresponding author upon reasonable request.
